# Circulating Vaccine Derived Poliovirus and the polio eradication endgame

**Published:** 2012-08-20

**Authors:** Robert Davis, Peter F Wright

**Affiliations:** 1 American Red Cross, Nairobi, Kenya; 2 Department of Pediatrics, The Geisel School of Medicine at Dartmouth, Lebanon, NH, USA

**Keywords:** Polio, polio eradication, vaccine derived poliovirus, Oral polio vaccine, inactivated polio vaccine, surveillance

## Editorial

The world is now in the 24^th^ year of the Global Polio Eradication Initiative. Halfway through that initiative in 2000, the island of Hispaniola saw an outbreak of vaccine derived poliovirus [1]. The implications of this outbreak and multiple subsequent isolations of Sabin strains that are estimated to have circulated in communities for a year or more are now causing a reconsideration of the optimal way to achieve polio eradication. These circulating vaccine derived polio viruses, cVDPV, in many cases have regained the virulence and capacity to cause paralytic disease that we associate with wild-type poliovirus (WPV) strains. They, with the prolonged poliovirus vaccine virus shedding seen in some immunodeficient children, iVDPV, have uncovered a serious risk to polio eradication using OPV as our sole weapon.

After the Hispaniola outbreak, retrospective studies of polio stool samples showed that a multiyear cVDPV epidemic had struck Egypt in the 1990s [2] and cVDPV type 2 has persisted for multiple years in Nigeria [3].

Shorter contained cVDPV outbreaks have occurred in many areas of the world, most typically in areas with inadequate vaccine coverage and low strain specific immunity. They have each been interrupted with a renewed effort at increasing OPV coverage. If safe and effective antivirals become available in the next few years, could they serve for ring containment of cVDPV or to treat the identified people with iVDPV?

### Surveillance issues, and the growing relevance of environmental sampling

New vaccination policies may make WPV and cVDPV, with case to infection ratios of 1:100 and higher, even harder to find than at present by classical AFP surveillance. IPV may prevent clinical illness without stopping transmission. Moreover, AFP surveillance may decline in the final years of eradication and, especially, post-eradication. In that case, will environmental sampling be needed on a larger scale? At present it is time-consuming and requires some at least rudimentary sewage system, but in Egypt it was key to documenting persistent cVDPV [3] and to eradication of WPV [4]. Environmental surveillance now serves as a useful check on the quality of AFP surveillance and as a tool for detecting iVDPV from immunodeficients. Its importance will grow in the post-eradication era.

### Oral Polio Vaccine, friend and enemy

The OPV paradox states that we produce cVDPV by vaccination and we prevent cVDPV by vaccination. The absence of wild type polio from India for over a year speaks to the power of OPV as a tool for polio eradication [5]. The paradox does not apply in areas where OPV coverage is high enough to prevent cVDPV introduction. An additional paradox is the rare but well established occurrence of paralytic disease as a direct result of OPV administration. These risks do not apply in countries which have used inactivated poliovirus vaccine (IPV) as their long-standing defense against polio or to the increasing number of countries which after achieving high OPV coverage have switched to IPV.

### Bivalent types 1 and 3 OPV (bOPV)

On WHO advice, many countries have adopted a mixed schedule, retaining trivalent types 1, 2, and 3 vaccine (tOPV) for routine and using bOPV for campaigns. While this strategy holds the promise of stopping types 1 and 3 WPV, it also carries risks. Some see continuing type 2 cVDPV transmission in Nigeria as linked to Nigeria's extensive use of bOPV in campaigns, a measure thought necessary to stop WPV transmission in Africa's only remaining polio endemic country, while inefficiently administering tOPV in the routine program. Once the world stops transmission of WPV types 1 and 3 (confined in 2012 to Chad and the three endemic countries of Nigeria, Afghanistan and Pakistan), the remaining problem of cVDPV, especially type 2, could be overcome, in the interim, by systematic and high quality coverage with tOPV or monovalent type 2 OPV. The importance of having an optimal type 2 OPV has led to an effort to develop a safer and more genetically stable type 2 strain.

### An OPV to IPV transition

For countries currently using OPV, several solutions to this problem have been proposed:

- Introduction of IPV into the EPI program, while continuing the use of tOPV or bOPV through the Global Polio Eradication Initiative (South Africa and several Latin American countries have recently done this)
- Cessation of type 2 OPV, which accounts for most cVDPV, while continuing vaccination with types 1 and 3, using bivalent OPV (bOPV). The bOPV option, while creating risks of type 2 cVDPV, produces higher seroconversion rates for types 1 and 3, both of which are still endemic as wild type viruses in the world.
- Discontinuation of all OPV, and continuation of IPV, once cessation of WPV and cVDPV is documented.

All of these solutions will be aided by development of easier and less expensive approaches to the use of IPV so that it can readily be used in mass campaigns. Discontinuation of all OPV is a necessary step on the critical pathway to polio eradication. An interim step of discontinuing all live type 2 vaccine is viewed as highly informative and the safest route to eradication.

### Inactivated Polio Vaccine, savior or mirage

Polio vaccination policy, especially in industrialized countries, has seen many transitions between OPV and IPV use. The US, for example, used IPV in the 50s, before going over to OPV for most vaccinees after 1961. A joint IPV/OPV regimen, used in the ‘90s, was abandoned in 2000 in favor of the straight IPV schedule now in use.

Early adopters of IPV (Sweden and the Netherlands, among others) pointed to the safety of the product, to its protection of the individual, and to its priming effect. IPV has stopped polio transmission in many countries; the 56 countries now using it exclusively have witnessed rapid disappearance of OPV from their environments. However, where challenges with OPV have been done in IPV vaccinated individuals, shedding of OPV has been detected in diminished but substantial amounts [6]. This OPV shedding in spite of systemic immunity induced by IPV is pertinent to concerns about possible cVDPV as countries make the OPV to IPV switch.

Individual countries and regions will make decisions about their polio immunization strategy. The 2012 World Health Assembly was a sounding board for reservations about IPV. Concerns about IPV included questions related to cost, mucosal immunity, and to herd immunity.

For most developing countries, the use of full-dose IPV by the intramuscular route is too expensive. WHO is now supporting research on intradermal administration of IPV through use of hand held injectors. This solution is at least two years from field implementation. Another question is how many doses of IPV would be needed to assure solid immunity. Recent work from Cuba points to a priming effect from a single dose IPV in previously naïve subjects (Resik S, unpublished data). Research on intradermal administration of IPV in fractional doses is informing policy making in this area [7, 8] Although IPV has eradicated polio in a number of European countries, we are learning from studies such as these that IPV does not provide as strong mucosal immunity against virus shedding as OPV. Further complicating the full endorsement of IPV is the manufacturing risks associated with production of large quantities of WPVs that are the components of the present IPV. A WHO-led initiative is preparing the introduction of IPV made from the attenuated Sabin strains.

An advocacy effort may be necessary to persuade reluctant governments that IPV introduction is a prerequisite for the OPV cessation endorsed in 2008 by the World Health Assembly (WHA Resolution 61.1) Only a few countries in Asia and Africa are current IPV users. For most countries, a technology needs final development and field testing which will permit safe and effective house to house administration of IPV, preferably in fractional doses. Building on research work, WHO and its member states will need to look at the schedule for IPV introduction and whether (especially in countries like Nigeria and the Democratic Republic of the Congo) a mass OPV or IPV campaign may be necessary to stop persistent cVDPV transmission. Also in development are antiviral drugs that might have a role in interrupting cVDPV circulation.

### Will a combined regime of OPV and IPV be the best of both worlds?

The highest immunity to all three polio types in developing country settings has been seen to a combined regimen of OPV and IPV [9]. A number of countries are employing mixed IPV and OPV though it seems an interim step. We are still asking the question of whether OPV vaccination can safely cease without well documented high levels of IPV coverage. Each country that switches from OPV to IPV is another experiment in the feasibility of doing this.

### Assumptions for cessation of Type 2 OPV

The world has detected no type 2 WPV since the last cases were seen in northern India in 1999. If bOPV schedules, with or without IPV, are adopted, then Sabin Type 2 will disappear from circulation. Here, there is a mixed picture, with continued circulation of 1 lineage in Nigeria, but apparent limited (rare) occurrence in other countries and prompt disappearance of many lineages with appropriate use of OPV containing Type 2.

A global consensus on dropping the type 2 component of OPV is being sought. If OPV 2 can be stopped, probably with IPV use, this will be instructive to the ultimate polio endgame in terms of reducing the risk of cVDPV and overall strategy. It would eliminate a major component of vaccine associated paralytic polio and eliminate the competition of type 2 with other OPV Sabin strains.

### Challenges of discontinuing all OPV, while continuing IPV

Once cessation of circulating VDPV and WPV have been well documented, routine IPV administration will continue in many countries, either as a public health or a national security measure. This will reinforce the need for efficient delivery systems for IPV. While combined vaccines exist which permit simultaneous administration of IPV with DPT, some of these include acellular pertussis vaccine, not accepted in most developing countries. There are product development and licensure issues here, which are likely to involve the GAVI Alliance, current provider of pentavalent vaccine to many African and Asian countries, as a major partner.

### Post-discontinuation of OPV

All countries must agree on the need for cessation of OPV, with sensitive surveillance to show the absence of WPV and cVDPV. The elimination of the type 2 OPV component and the switch to IPV require both global policy guidance and consultations with all of the polio community.

Once GPEI, like the Smallpox Eradication Programme before it, ceases to exist, the world must build on its accomplishments. As the world turns its attention towards measles and rubella issues, the surveillance and laboratory networks created for GPEI can serve broader purposes, including, in many countries, integrated disease surveillance and response (IDSR). House to house strategies developed by GPEI can serve other initiatives as well. The AFP surveillance strategies should not collapse. In particular, post-eradication surveillance should demonstrate the decline in AFP cases with residual paralysis.
